# Bilateral skeletonized IMA: in situ grafts for myocardial revascularization

**DOI:** 10.1186/1749-8090-8-S1-O184

**Published:** 2013-09-11

**Authors:** Z Jonjev, M Rosic, S Majdevac, M Fabri

**Affiliations:** 1 Institute for CVD of Vojvodina, Sremska Kamenica, Serbia

## Background

Bilateral skeletonized internal mammary artery (IMA) has been recognized as the most advanced surgical option for myocardial revascularization. However, diabetes, chronic obstructive pulmonary disease (COPD) and obesity have been accepted as a limitation for such surgical option. The aim of this study is to evaluate immediate and long term results in patients with bilateral skeletonized IMA used as “in situ” graft for coronary bypass surgery.

## Methods

We prospectively analyzed 110 patients operated between 2003 - 2013 with multivessel coronary artery disease. All patients were operated on as elective cases. Most of the patients were male (84.54%), with average age 56.17 years (33-75 y/o). Eighteen patients were diabetic (15.84%), and 28 (24.75%) have had COPD. The average number of grafts was 2.75, and average ejection fraction was 50.74% (Range=25-65%).

## Results

Despite relatively high preoperative Euro and STS score there was no postoperative mortality (30 days). There were no perioperative myocardial infarctions or cerebrovascular incidence. One patient had minor presternal wound infection. The average length of hospital stay was 8 days.

## Conclusion

Bilateral skeletonized IMA could be successfully used as a conduit for CABG, especially if total arterial revascularization is preferred. IMA harvesting with skeletonized technique provides better IMA length, detailed graft visualization, and minimal trauma to the chest wall. That makes traditionally accepted limitations for usage of bilateral IMA (i.e. diabetes and COPD) irrelevant.

